# HiFi based metagenomic assembly strategy provides accuracy near isolated genome resolution in MAG assembly

**DOI:** 10.1002/imo2.70041

**Published:** 2025-07-08

**Authors:** Feilong Deng, Yanhua Han, Minghui Li, Yunjuan Peng, Jianmin Chai, Guan Yang, Ying Li, Jiangchao Zhao

**Affiliations:** ^1^ Guangdong Provincial Key Laboratory of Animal Molecular Design and Precise Breeding School of Animal Science and Technology, Foshan University Foshan China; ^2^ School of Animal Science and Technology, Foshan University Foshan China; ^3^ Guangdong Laboratory for Lingnan Modern Agriculture, Animal Functional Microbiome Lab College of Animal Science, South China Agricultural University Guangzhou China; ^4^ Department of Infectious Diseases and Public Health City University of Hong Kong Hong Kong China

## Abstract

Recovering high‐contiguity, circular bacterial genomes from complex microbiomes (e.g., gut) is challenged by limitations of short‐read and error‐prone long‐read sequencing. This study comprehensively compares PacBio High‐Fidelity (HiFi) sequencing‐based metagenome‐assembled genomes (MAGs) against Illumina MAGs, Oxford Nanopore Technologies (ONT) MAGs, and isolate whole‐genome sequencing genomes from the same sample. HiFi sequencing yielded 31 high‐quality MAGs, including 10 complete circular genomes. HiFi MAGs demonstrated significantly higher completeness, continuity, and lower contamination than Illumina or ONT MAGs (*p‐*adj < 0.05). Crucially, HiFi MAGs exhibited closer genomic proximity to corresponding isolates at both single‐nucleotide polymorphism and gene presence/absence levels. This benchmarking establishes HiFi as a robust approach for generating MAGs rivaling isolated genome quality, providing critical insights for accurate microbial genomic studies.

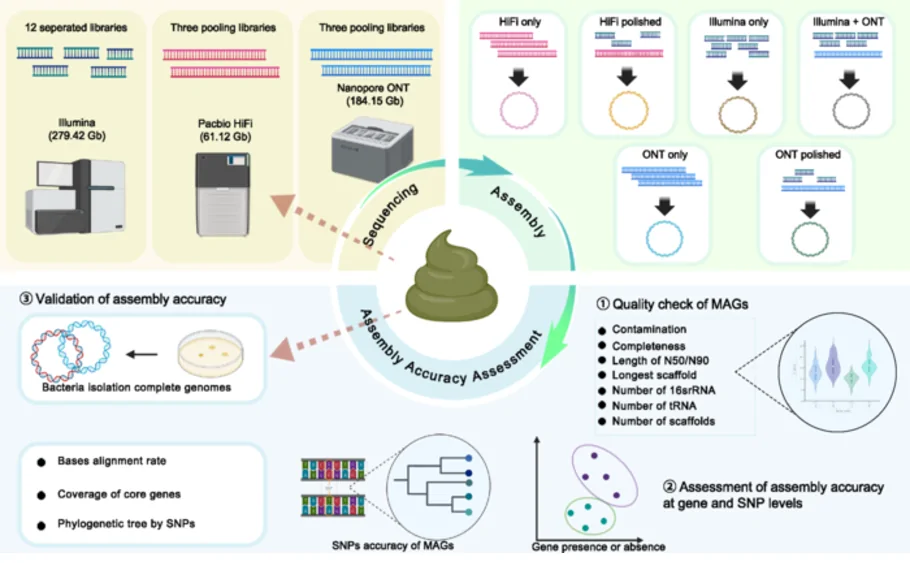

To the Editor,

High‐quality reference genomes are crucial for exploring microbial diversity and functions in various micro‐ecological environments. A major challenge is the lack of reference genomes for a large number of unculturable microbiota. Metagenome‐assembled genomes (MAGs) have become a key resource to fill this gap, as exemplified by their extensive incorporation into major reference databases. For instance, the Genome Taxonomy Database (GTDB Release 226) currently documents 715,230 bacterial species, a substantial proportion of which derived from MAG‐based analyses [1].

Recent research in the scientific community has focused on the reconstruction of MAGs from various environments, including animal gut microbiome [2, 3] and marine ecosystems [4]. The effectiveness of these MAGs is often limited by the varying assembly quality of traditional short‐read sequencing methods and the higher error rates of third‐generation technologies [5, 6]. Pacific Biosciences (PacBio) has advanced the field with its precise long‐read sequencing technology, addressing the shortcomings of previous methods and may improve metagenomic assembly quality [7, 8]. Previous comparative studies have primarily evaluated MAGs based on metrics such as contiguity, completeness (including rRNA and tRNA integrity), and contamination [9, 10]. Kim et al. [11] demonstrated that HiFi‐derived MAGs exhibited >99.99% average nucleotide identity and alignment coverage when compared to reference genomes from NCBI/REF databases. However, their studies lacked direct methodological comparisons and relied on public database‐derived reference genomes, which may not fully represent intraspecies strain diversity [11]. This study compared MAGs obtained from the same samples using Illumina, Oxford Nanopore Technologies (ONT), and HiFi sequencing, aiming to identify differences in assembly quality. By isolating and sequencing microorganisms from these samples to create a benchmark genomic map, we assessed the fidelity of different sequencing methods in accurately replicating genomes, offering insights into the effectiveness of current metagenomic assembly techniques and their practical applications.

## 1 RESULTS AND DISCUSSION

### Summary of data and assembly of MAGs

To perform a comprehensive comparison, a total number of 8.45 M HiFi reads were successfully generated, collectively spanning a cumulative data depth of 61.12 Gb, and exhibiting an average read length of 7232 bp (Table S1). Moreover, Illumina sequencing data (279.42 Gb) and nanopore sequencing data (184.15 Gb) from our previous study were assimilated into this study [2]. The Illumina sequencing data, nanopore sequencing data, and PacBio HiFi data were derived from the same set of samples (Table S1). Three HiFi pool samples were assembled independently utilizing Flye software, resulting in a cumulative total of 31 nonredundant MAGs with high‐quality (completeness of ≥90% and contamination <5%). Among the high‐quality MAGs, 10 were identified as accurate and complete MAGs, each exhibiting a genomic circularity, and additional seven MAGs each had a singular scaffold.

We further assembled the 12 Illumina sequencing samples independently. A total of 57 high‐quality MAGs were included; this larger MAG number is attributable to the significantly higher sequencing depth allocated to the Illumina platform. For the ONT data set, only 14 high‐quality MAGs were identified from ONT assembled MAGs. In our previous study [12], we constructed MAGs with hybrid methods integrating Illumina and ONT data. Sixty‐one high‐quality MAGs were achieved and used for downstream analysis. Considering the raw sequencing error rates of ONT (~10% to 15%) [13] and HiFi (~1%) [14, 15], we refined ONT and HiFi long reads utilizing the Illumina reads. Thirty‐three MAGs derived from polished HiFi and 17 from polished ONT reads were qualified as high‐quality and were subsequently incorporated into the analysis (Table S2). Here, sequencing depths were not uniformly maintained across platforms. Standardizing depths and assembly parameters for Illumina/ONT to match HiFi specifications would have compromised high‐quality MAG recovery, especially from Illumina data. To enable assembly accuracy comparisons at single‐nucleotide polymorphism (SNP) and gene levels using isolated single‐strain reference genomes, we prioritized retaining MAGs critical for cross‐strategy comparisons over depth normalization. Thus, Illumina and ONT sequencing depths and assembly parameters were optimized to maximize MAG yield without compromising comparative analyses.

### Quality comparison of MAGs by different assembly methods

**Figure 1:**
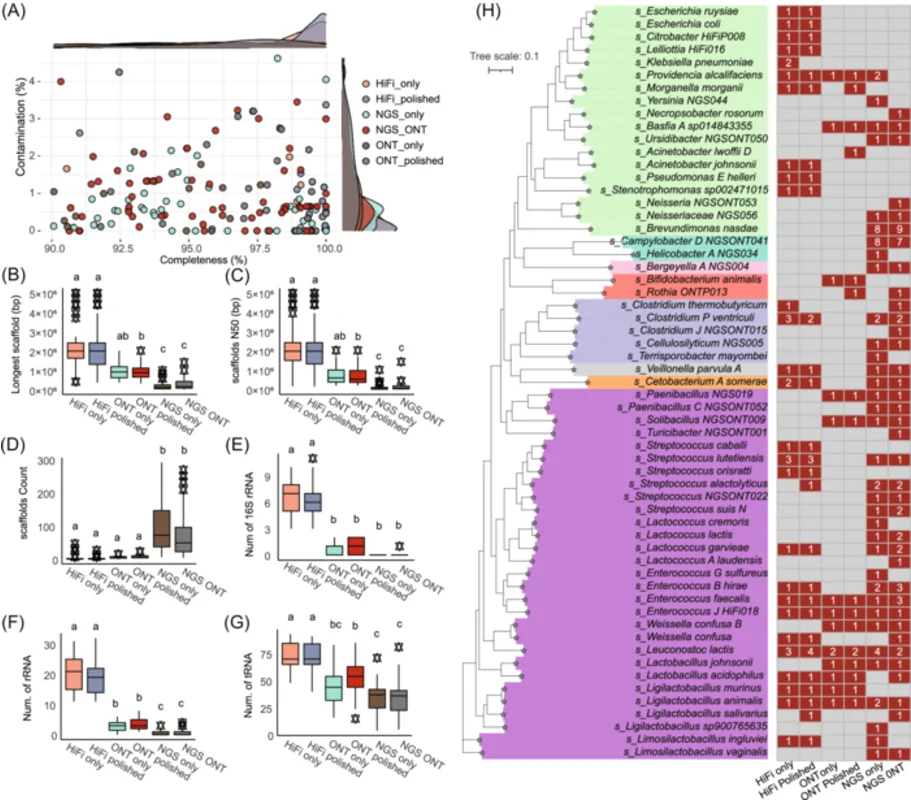
Comparative analysis of metagenome‐assembled genomes (MAGs) quality across assembly methods. This figure presents a detailed comparison of MAGs assembled using various methods, highlighting aspects such as contamination and completeness (A), the length of the longest scaffold (B), N50 scaffold length (C), total scaffold count (D), the count of 16S rRNA genes specifically (E), the overall number of rRNA genes (F), and the number of tRNA genes (G). Panel (H) displays the phylogenetic tree of all assembled MAGs (species), with a heatmap indicating the number of MAGs per assembly method. Gray cells represent the absence of high‐quality MAG, while red cells indicate their presence, with numbers quantifying the count per method. The tree's clade backgrounds are color‐coded to represent different bacterial phyla, illustrating the diversity captured in the assemblies. The phylogenetic tree was constructed using PhyloPhlan (v3.0.2) and visualized with the Interactive Tree of Life (iTOL, v6.5.2). Group comparisons were conducted using Kruskal–Wallis tests, with Dunn's post hoc analysis incorporating Benjamini–Hochberg false discovery rate correction (*α* = 0.05). Significant differences (*p*‐adj < 0.05) are indicated by unique superscript letters (a–c). Groups with identical letters show no significant differences.

Evaluations using CheckM software revealed that HiFi‐based assemblies (HiFi‐only and HiFi‐polished) achieved significantly higher completeness than ONT‐based (ONT‐only and ONT‐polished) and Illumina‐based groups (Next‐Generation Sequencing, NGS‐only and NGS‐ONT hybrid) (*p‐*adj < 0.05, Benjamini‐Hochberg FDR‐adjusted *p*‐value, Figure 1A). Contamination was also significantly reduced in HiFi‐based groups relative to ONT‐polished and NGS‐ONT hybrid assemblies (*p‐*adj < 0.05), though not statistically distinct from ONT‐only or NGS‐only groups (*p‐*adj ≥ 0.05). Additionally, HiFi‐based assemblies (HiFi‐only and HiFi‐polished) demonstrated significantly longer scaffold lengths (Longest and N50) than all other groups except ONT‐only (*p‐*adj < 0.05, Figure 1B,C), indicating superior integrity and continuity. In terms of scaffold count, NGS‐only and NGS‐ONT groups exceeded others, highlighting a disparity in scaffold abundance (*p‐*adj < 0.05, Figure 1D). Importantly, all high‐quality MAGs assembled with HiFi‐based methods contained at least one 16S rRNA gene. The HiFi‐only and HiFi‐polished groups had notably higher counts of 16S rRNA gene compared to their counterparts (*p‐*adj < 0.05, Figure 1E), which was consistent with Tao et al.'s study [7, 9], emphasizing HiFi's effectiveness in capturing 16S rRNA sequence. This significant difference in 16S rRNA gene prevalence and the aggregate counts of rRNA and tRNA across groups are depicted in Figure 1E–G. Comparing multiple assembly strategies, we observed that both HiFi and ONT‐based methods significantly improve the length and continuity of MAGs. These methods also reduced assembly contamination and enhanced the completeness of assemblies, including an increased number of assembled tRNA genes. The advantage of ONT on MAGs assembly has been confirmed by a previous study [16]. Notably, the HiFi strategy was particularly effective in assembling 16S rRNA genes, while the ONT‐based strategy yielded limited success in this regard. The higher error rate associated with ONT might be a primary factor contributing to its challenges in assembling highly conserved regions, such as 16S rRNA gene.

### Assessment of assembly accuracy at gene and single‐nucleotide resolution

**Figure 2:**
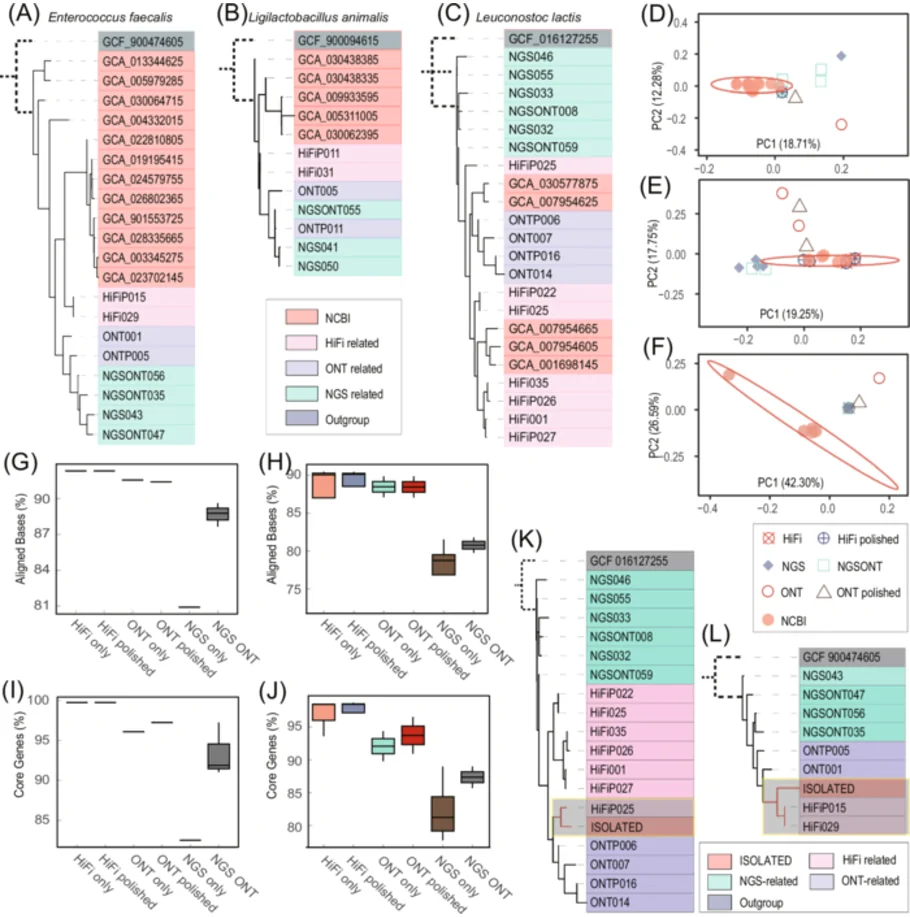
Assessment of metagenome‐assembled genome (MAG) assembly accuracy using various sequencing technologies based on comparison with isolate genomes. Panels (A–C) show the phylogenetic trees of MAGs and isolate genomes from NCBI for *Enterococcus faecalis* (A) and *Ligilactobacillus animalis* (B), along with *Leuconostoc lactis* (C). Panels (D–F) present the principal coordinates analysis (PCoA) plots based on the Jaccard distance matrix concerning the gene presence or absence in MAG/genomes for *Enterococcus faecalis* (E), *Leuconostoc lactis* (E), and *Ligilactobacillus animalis* (F). The alignment rate of MAGs' bases and core gene coverage for *Enterococcus faecalis* (G, H) and *Leuconostoc lactis* (I, J) are shown in boxplots in Panels (G–J). Panels (K) and (L) depict the phylogenetic trees of MAGs and isolate genomes from giant pandas for *Leuconostoc lactis* (K) and *Enterococcus faecalis* (L).

A total of 217 high‐quality MAGs from six assembly groups were classified into 59 distinct clusters with 95% similarity, comprising 41 known and 18 potential novel species (Figure 1H). Remarkably, only four species, including *Ligilactobacillus animalis*, *Leuconostoc lactis*, *Enterococcus faecalis*, and a novel *Enterococcus* species (*Enterococcus J HiFi018*), were consistently assembled across all methodologies (Figure 1H). In general, the primary types of errors in metagenomic assembly of genomes include: the deletion or addition of genomic fragments, and secondly, base errors at the single‐nucleotide level [17]. To assess assembly accuracy, we compared the MAGs of *Leuconostoc lactis, Enterococcus faecalis*, and *Ligilactobacillus animalis* with genomes identified as “Complete Genome” in the NCBI database. Phylogenetic analysis revealed that HiFi‐based MAGs were closer aligned with complete NCBI genomes for three of *E. faecalis* (Figure 2A) and *L*. *animalis* (Figure 2B). For *L. lactis* (Figure 2C), ONT‐based MAGs and HiFi‐based MAGs are each located in the same evolutionary branch with different strains of *L. lactis* from the NCBI database. The results indicate that HiFi long reads may offer advantages over Illumina reads in assembling MAGs at the SNP level. Although Illumina sequencing technology has higher sequencing accuracy than HiFi or ONT sequencing technologies [18], it does not necessarily translate to increased accuracy in MAG assembly. SNPs located within homologous gene fragments can mutually interfere during the assembly process, leading to errors at the SNP level. This issue arises when the length of these homologous gene fragments surpasses the length of the sequencing reads, rendering the assembly software unable to distinguish between the SNPs. In contrast, long reads are capable of accurately assigning SNPs between homologous fragments to the correct MAGs.

The detection of structural variants using the HiFi method has been employed in studies of eukaryotic organisms [19]. Here, the principal coordinates analysis (PCoA) based on gene presence/absence showed HiFi MAGs were closer to NCBI genomes than those from other groups for both *E. faecalis* (Figure 2D) and *L*. *lactis* (Figure 2E). For the *L. animalis* (Figure 2F), the Illumina‐ and HiFi‐based MAGs almost overlap on the PCoA plot. The results showed that HiFi sequencing method also does good performance on integrality of MAGs assembly and suggested that HiFi‐based MAGs are well‐suited for detecting gene transfer and absence, a critical aspect in comparative genomics research. Given the dynamic nature of microbial genomes, which involves extensive horizontal gene transfer and gene loss, HiFi sequencing provides an essential tool for understanding these complexities. No significant difference was found between HiFi‐only and HiFi‐polished MAGs, indicating Illumina data polishing does not markedly alter HiFi gene assembly.

In this study, due to the absence of reference genomes from gut microbiome of giant pandas, strain variations within the same species were not accounted for. We overcame this limitation by isolating and culturing *L. lactis* and *E. faecalis* from the samples previously used in high‐throughput sequencing, and subsequently performing their whole‐genome sequencing. These isolated genomes were treated as the “gold standard” for comparison with MAGs. The genomic base alignment rate for HiFi‐based MAGs was notably higher than that of other methods for both *E. faecalis* (Figure 2G) and *L*. *lactis* (Figure 2H). At the gene level, the core gene coverage of HiFi‐based MAGs surpassed that of other methods (Figure 2I,J). Phylogenetic analyses revealed that at least one MAG assembled via the HiFi method clustered in the same branch (Figure 2K,L), confirming the superior assembly accuracy of the HiFi method compared to ONT‐ or Illumina‐based methods. Yet again, here, we demonstrated that the HiFi sequencing approach enhances gene completeness and base‐level accuracy in the assembly of MAGs.

While our study has yielded several significant conclusions, it's important to acknowledge certain limitations. Firstly, there was a disparity in the total volume of sequencing data among the Illumina, ONT, and HiFi methods, with Illumina having the highest volume, followed by ONT, and HiFi having the least. Despite this, our findings indicated that HiFi achieved the most optimal assembly results with less data across multiple dimensions. Secondly, although we isolated *E. faecalis* and *L. lactis* from the samples as a “gold standard” for comparison, considering the mixed nature of our samples, the diversity within species could mean that the assembled MAGs and the isolated strains inherently differ [20].

## 2 CONCLUSION

In conclusion, this study presents a comparative analysis of metagenomic assemblies utilizing Illumina, ONT, and HiFi sequencing technologies. The investigation primarily focuses on evaluating assembly completeness, contamination, genomic continuity, and accuracy at both the single‐nucleotide and gene levels. The findings demonstrate that the HiFi metagenomic assembly method achieves a level of precision approaching that of isolated genomes derived from the same sample. This highlights the potential of HiFi metagenomics for future research endeavors that require highly accurate microbial genomic data.

## 3 METHODS

Detailed procedures for materials and methods are available in the Supplementary Information.

## AUTHOR CONTRIBUTIONS

**Feilong Deng**: Writing—original draft; methodology; software; visualization; funding acquisition. **Yanhua Han**: Investigation; visualization. **Minghui Li**: Visualization. **Yunjuan Peng**: Investigation. **Jianmin Chai**: Visualization. **Guan Yang**: Writing—review & editing. **Ying Li**: Writing—review & editing; conceptualization; funding acquisition. **Jiangchao Zhao**: Conceptualization; funding acquisition; writing—review & editing; supervision.

## CONFLICT OF INTEREST STATEMENT

The authors declare no conflicts of interest.

## ETHICS STATEMENT

The ethics application (No. FOSU2020002) was approved by the Animal Care and Use Committee of Foshan University.

## Supporting information


**Table S1.** Summary of Cleaned Sequencing Data from Illumina, Nanopore, and PacBio Platforms.
**Table S2.** Assessment and Taxonomy of High‐Quality MAGs Across Assembly Methods.

## Data Availability

The sequence data generated in this study have been deposited in the NCBI Sequence Read Archive (SRA) database and are accessible through the accession numbers https://www.ncbi.nlm.nih.gov/bioproject/PRJNA872265 for Illumina reads and https://www.ncbi.nlm.nih.gov/bioproject/PRJNA1073587 for ONT and PacBio long reads. The metadata for all samples included in this study are detailed in Supplementary Table S1. Bioinformatic analysis pipeline was deposited in the GitHub (https://github.com/FdengMicrobiome/-HiFi-MAG-Assembly/wiki/HiFi%E2%80%90Driven-MAGs-Assembly-Workflow). Supplementary materials (methods, tables, graphical abstract, slides, videos, Chinese translated version, and update materials) may be found in the online DOI or iMeta Science http://www.imeta.science/imetaomics/.
